# Identification of specific role of SNX family in gastric cancer prognosis evaluation

**DOI:** 10.1038/s41598-022-14266-y

**Published:** 2022-06-17

**Authors:** Beibei Hu, Guohui Yin, Xuren Sun

**Affiliations:** 1 Department of Gastroenterology, First Affiliated Hospital of China Medical University, North Nanjing Street 155, Shenyang, 110001 People’s Republic of China; 2 School of Civil Engineering and Transportation, Hebei University of Technology, Tianjin, 300401 People’s Republic of China

**Keywords:** Computational biology and bioinformatics, Gastroenterology, Oncology

## Abstract

We here perform a systematic bioinformatic analysis to uncover the role of sorting nexin (SNX) family in clinical outcome of gastric cancer (GC). Comprehensive bioinformatic analysis were realized with online tools such as TCGA, GEO, String, Timer, cBioportal and Kaplan–Meier Plotter. Statistical analysis was conducted with R language or Perl, and artificial neural network (ANN) model was established using Python. Our analysis demonstrated that SNX4/5/6/7/8/10/13/14/15/16/20/22/25/27/30 were higher expressed in GC, whereas SNX1/17/21/24/33 were in the opposite expression profiles. GSE66229 was employed as verification of the differential expression analysis based on TCGA. Clustering results gave the relative transcriptional levels of 30 SNXs in tumor, and it was totally consistent to the inner relevance of SNXs at mRNA level. Protein–Protein Interaction map showed closely and complex connection among 33 SNXs. Tumor immune infiltration analysis asserted that SNX1/3/9/18/19/21/29/33, SNX1/17/18/20/21/29/31/33, SNX1/2/3/6/10/18/29/33, and SNX1/2/6/10/17/18/20/29 were strongly correlated with four kinds of survival related tumor-infiltrating immune cells, including cancer associated fibroblast, endothelial cells, macrophages and Tregs. Kaplan–Meier survival analysis based on GEO presented more satisfactory results than that based on TCGA-STAD did, and all the 29 SNXs were statistically significant, SNX23/26/28 excluded. SNXs alteration contributed to microsatellite instability (MSI) or higher level of MSI-H (hyper-mutated MSI or high level of MSI), and other malignancy encompassing mutation of TP53 and ARID1A, as well as methylation of MLH1.The multivariate cox model, visualized as a nomogram, performed excellently in patients risk classification, for those with higher risk-score suffered from shorter overall survival (OS). Compared to previous researches, our ANN models showed a predictive power at a middle-upper level, with AUC of 0.87/0.72, 0.84/0.72, 0.90/0.71 (GSE84437), 0.98/0.66, 0.86/0.70, 0.98/0.71 (GSE66229), 0.94/0.66, 0.83/0.71, 0.88/0.72 (GSE26253) corresponding to one-, three- and five-year OS and recurrence free survival (RFS) estimation, especially ANN model built with GSE66229 including exclusively SNXs as input data. The SNX family shows great value in postoperative survival evaluation of GC, and ANN models constructed using SNXs transcriptional data manifesting excellent predictive power in both OS and RFS prediction works as convincing verification to that.

## Introduction

According to the latest report covering 185 countries including 36 cancer types, with breast cancer having become the most prevailing carcinoma worldwide and following by lung, colorectal and prostate, gastric cancer has already soared up to be the fifth most common cancer^1^. Besides, authority democratic statistical analysis revealed that the age-standardized incidence rate of GC had shown a stable decreasing tendency during the last three decades from 1990 to 2019 in China, while the numbers of newly occurring cases and the death will still increase to 738.79 thousand and 454.80 thousand in the next 25 years, saying that GC remains a non-negligible medical burden for both the world community and China^2^. For resectable GC, D2 lymphadenectomy is the only effective therapeutic strategy, but the five-year survival rate of them reaches only 20–30% once found metastatic lymph nodes positive. Therefore, searching for prognostic factors, including 18-FDG-PET assessment, EBV definition, mismatch repair (MMR) and microsatellite instability (MSI), and histological or pathological tumor response, can greatly help to fully assess the perioperative status of patients and determine the most suitable treatment for patients with resectable gastric cancer^3^.

The sorting nexin (SNX) family is a highly conserved protein family with PX domain, which specifically binds to phosphatidylinositol. To our knowledge, 33 SNX family members have been found in mammals, and SNX23/26/28 is also known as KIF16B/ARHGAP33/NOXO1^4^. According to the functional domains of SNXs, they can be classified into three types: SNX-PX, with the PX domain; SNX-PX-Bar, with both PX and Bar domains, such as SNX1/2/5/6/32; and SNX-PX-X, SNXs with both PX domain and other domains, such as SNX27 containing PDZ and FREM domains. Prior to illustrating the most important physiological function of the SNX family, we'd better first give a glimpse into the structure and function of the Retromer complex. The endomembrane system is unique to eukaryotes and consists of endoplasmic reticulum, golgi bodies, lysosomes, and various transporter vesicles. The endosomal network is a networked transport system consisting of numerous vesicles connected to the plasma membrane. After endocytosed into the endosomal system, cargo proteins are either sorted into lysosomes for degradation to downregulate signal transduction, or returned back to the trans golgi network (TGN) or cytomembrane for recycling. The retromer complex is responsible for sorting and transporting cargo proteins, acting as a critical role in intracellular biosynthesis and material secretion^5^. Retromer consists of two complex, cargo-recognition complex—VPS226/29/35; and cargo-selective complex—SNXs heterodimer, referring to SNX—Bar family in mammals. After the cargo proteins enter into the endosome system, the cargo-selective complex of Retromer will bind to the early endosomes, and with SNX-Bar's function of sensing membrane deformation, the binding region will deform and sag to form a smaller vesicle containing specific cargo proteins, which will then participate in the next transportation^6^. Aberrant expression or epigenetic modification of SNXs will lead to abnormal distribution of cargo proteins in the inner cell or on cell surface, thus to initiate or aggravate diseases. It has been reported that interaction of SNX1 and Enterophilin-1 decreased the distribution of epithelial growth factor receptor (EGFR) on cell surface^7^. Overexpression of SNX5 was documented to inhibit intracellular degradation of EGFR, while in hepatocellular carcinoma (HCC), SNX5 was witnessed to activate the EGFR-ERK1/2 pathway by lowering the circulation rate of EGFR in the endosomal network which leads to an increased amount of EGFR^8,9^. What's more, SNX1 has been reported to be a tumor suppressor in gastric cancer^10^. Whereas, little is known about values of other 32 SNX family members in GC prognosis until now.

With the development of computer technology, artificial intelligence technology, also known as AI, has permeated into various fields, including the construction of prediction models in cancers. So far, prediction models based on artificial intelligence has been employed in the diagnosis of precancerous status (chronic atrophic gastritis), prediction of the number of metastatic lymph nodes, histopathological diagnosis of GC, determination of stage, determination of inhibition of cell growth IC50, and long-term survival prediction and so on^11–16^. In other words, the application of artificial intelligence to construct prognostic models for predicting the overall survival accounts for only a tiny part when it comes to its usage in GC field. In this study, in order to further illustrate the significance of SNX family in the prognosis of GC, including OS and RFS, we combine clinical data and translational information of the SNX family, and use ANN to build prediction models aiming at one-, three-, and five-year survival prediction based on clinical data of three cohorts from South Korea and America.

In this study, we first explored the translational level of 30 members in SNX family (SNX23/26/28 not recorded), which was also proved to be a critical role of in GC prognosis by Kaplan-Meir survival analysis based on GEO mRNA data. Mutation analysis using cBioportal database indicated that alteration of SNX family exerted great impact on MSI occurrence in GC, and that those with SNX alteration were on a higher trend to undergo tumor-suppressing gene mutation, such as TP53, ARID1A, and MLH1.Clinical information from TCGA database and several SNXs shown as independent risk factors was employed to construct a multivariate cox regression model visualized as nomogram for OS prediction and risk classification. While other nine models using ANN based on clinical information from GEO encompassing GSE84437, GSE26253 and GSE66229 were established for OS and DFS prediction as verification of the TCGA nomogram model.

## Materials and methods

### The SNX family expressing profiles

**Figure 1:**
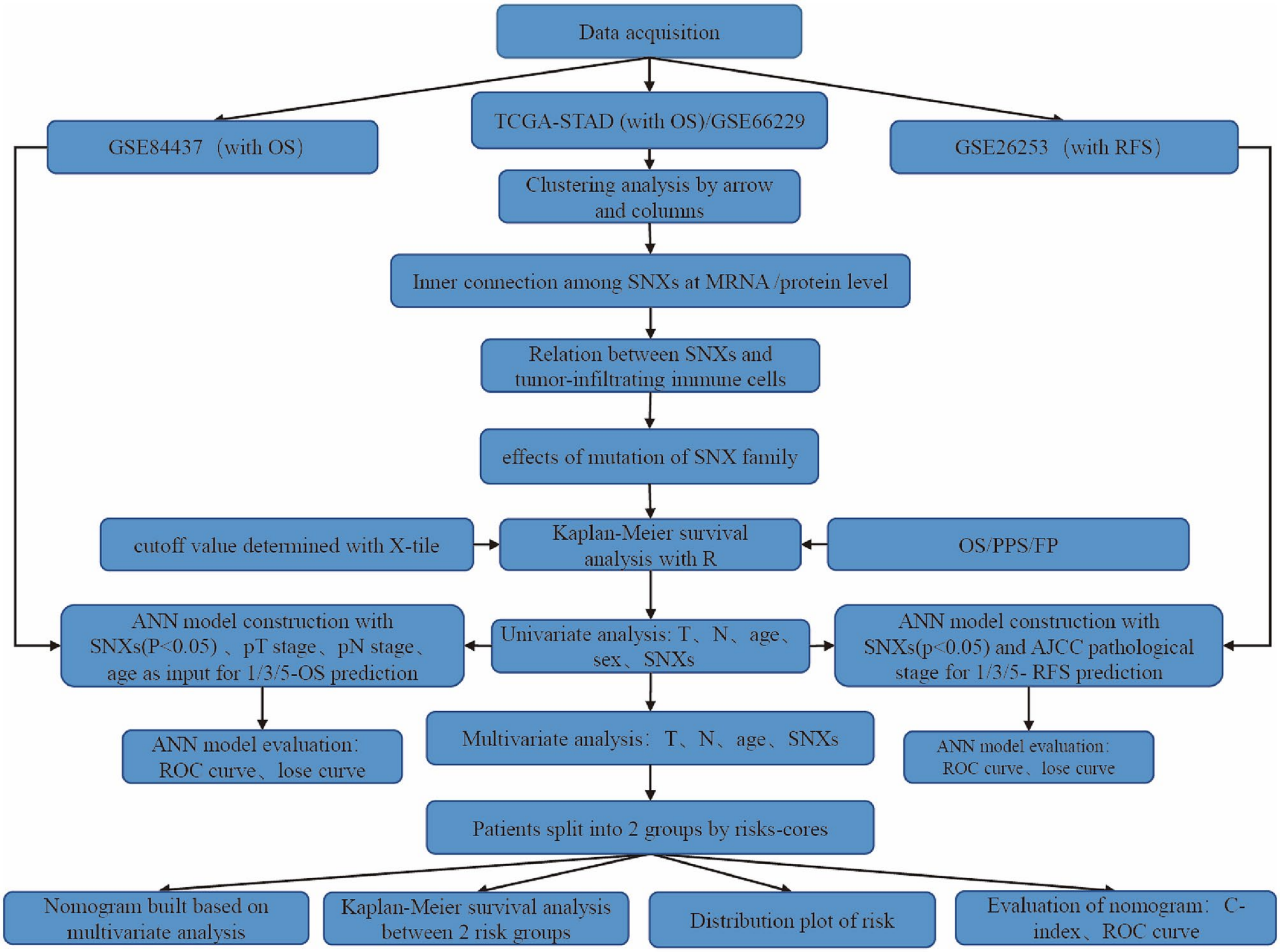
Workflow of this study. STAD, stomach adenocarcinoma; SNX, sorting nexin; OS, overall survival; RFS, relapse-free survival; PPS, post progression survival; PFS, progression-free survival.

Using perl language, TCGA-STAD transcriptome RNA-seq data on TCGA (https://portal.gdc.cancer.gov/) from 407 samples was downloaded, including tumorous tissue from 375 patients and paired non-cancerous mucosa from 32 patients. With no record of SNX23/26/28 displayed in the sequencing data, we only analyzed the mRNA level of other 30 SNXs in GC compared with the paired normal tissue. Besides, GSE66229 was also included for differential expression analysis as validation. Limma, the R package, was employed to perform the differential analysis and adjusted-*p* value < 0.05 was considered characteristic significant. Meanwhile, the result was visualized by the package beeswarm, with black and red referring to the normal and tumor group, respectively. To get a more intuitional glimpse of differential mRNA expression of SNXs between normal and tumor, 407 specimens were grouped into two and clustering analysis was also conducted with pheatmap R package. Workflow of this study was shown in Fig. 1.

### Inner correlation between SNXs at mRNA and protein levels

In this part, we first explored the internal correlation among 30 SNXs at mRNA level with igraph and reshape2 R packages, excepting for SNX23/26/28. Red and blue color referred to positive and negative correlation, respectively. String database (http://string-db.org/) provided protein–protein interaction map of 33 SNX members, and connecting lines between proteins stood for diverse channels from which the interaction was found such as literature searching, experiments and so on.

### Tumor immune infiltration analysis in TIMER database

TIMER is a comprehensive resource for systematic analysis of immune infiltrates across diverse cancer types (https://cistrome.shinyapps.io/timer/). Firstly, we drawn the KM curve of cumulative survival rate between the low and high TIICS infiltrating levels to find survival associated TIICs in GC. Then, Spearmen relevance analysis was applied to estimate correlation between SNXs and those TIICs.

### Mutation analysis in cBioportal database

The database cBioPortal (www.cbioportal.org) was employed to investigate effects of SNX family mutation. We analyzed the genomic profiles of 33 SNXs family members, encompassing mutation frequency, MSI associated items such as molecular subtype of GC, MSI Sensor score, MSI status and hyper-mutated, mutation of tumor suppressing genes like TP53, ARID1A and MLH1, as well as estimated lymphocyte percentage reflecting immune infiltration.

### Survival analysis and construction of risk classification model

Clinical characteristics including sex, age, T-stage, N-stage of 346 GC patients was downloaded from TCGA database and involved in the later survival analysis, 29 specimens excluded because of incomplete following-up information. With help of X-tile software, the best cut-off value was determined and 346 specimens was classified into two groups with low or high level of SNXs mRNA expression. Then, Kaplan–Meier curve of cumulative survival rate was plotted using two R packages named survmine and survival, and cut-off value of P value was set as 0.05. Kaplan–Meier Plotter (www.kmplot.com) was later used for prognostic role of SNX family in OS, progression-free survival (PFS), and post progression survival (PPS) of patients with gastric cancer. Additionally, univariate and multivariate cox regression analysis were performed in searching for risk factors of GC based on TCGA clinical information, and the results were visualized with forest plots. Finally, a nomogram based on multivariate cox regression model for one-/three-/five-year OS prediction was plotted with rms and foreign R packages. With pheatmap R package, risk-score, survival time and mortality of all the patients were displayed with risk distribution plots. What’s more, ROC curve of was also employed to estimate the accuracy of the nomogram using survivalROC package. GSVA, limma and GSEABase were used for single sample gene set enrichment analysis(ssGSEA), and difference in immune scores between high/low risk-score group was visualized with Limma, ggpubr and reshape2 packages, *P* < 0.05 considered statistic significant.

### Artificial neural network for prognosis model construction

Artificial neural network, ANN, is a major part of deep-learning affiliated to artificial intelligence. In this part, GSE84437 and GSE26253 of two Korea cohorts were downloaded from the Gene Expression Omnibus (GEO) database (https://www.ncbi.nlm.nih.gov/geo/), and used for model construction aiming at one -/three -/ five-year OS and RFS prediction. Therefore, six ANN models with four layers of neurons were constructed as verification of the TCGA nomogram model, including one input layer, one output layer and two hidden layers. Finally, GSE66229 with 300 GC samples from USA was also included for an ANN model for OS prediction, with only SNXs as input and thus working as more convincing proof of prognostic role of SNX family in GC. To ensure the generalization ability of the model, appropriate regularization was added to the hidden layer to prevent the over-fitting of the model. In order to get the best model for OS and RFS prediction, hyperparameters of each model was optimized, including neurons number in each hidden layer, regularization options of each hidden layer such as weight regularization, output regularization and bias regularization, types of optimizers of the output layer such as Adam and Rmsprop as well as batch data size. GSE84437, GSE66229 and GSE26253 were divided by a ratio of 7:3, 7/10 for training and 3/10 for validation. The number of iterations kept going up until loss of training sets stopped falling. Construction and optimization of ANN models were realized under the language environment of Python 3.5.

## Results

### Transcriptional level of SNX family in GC

**Figure 2:**
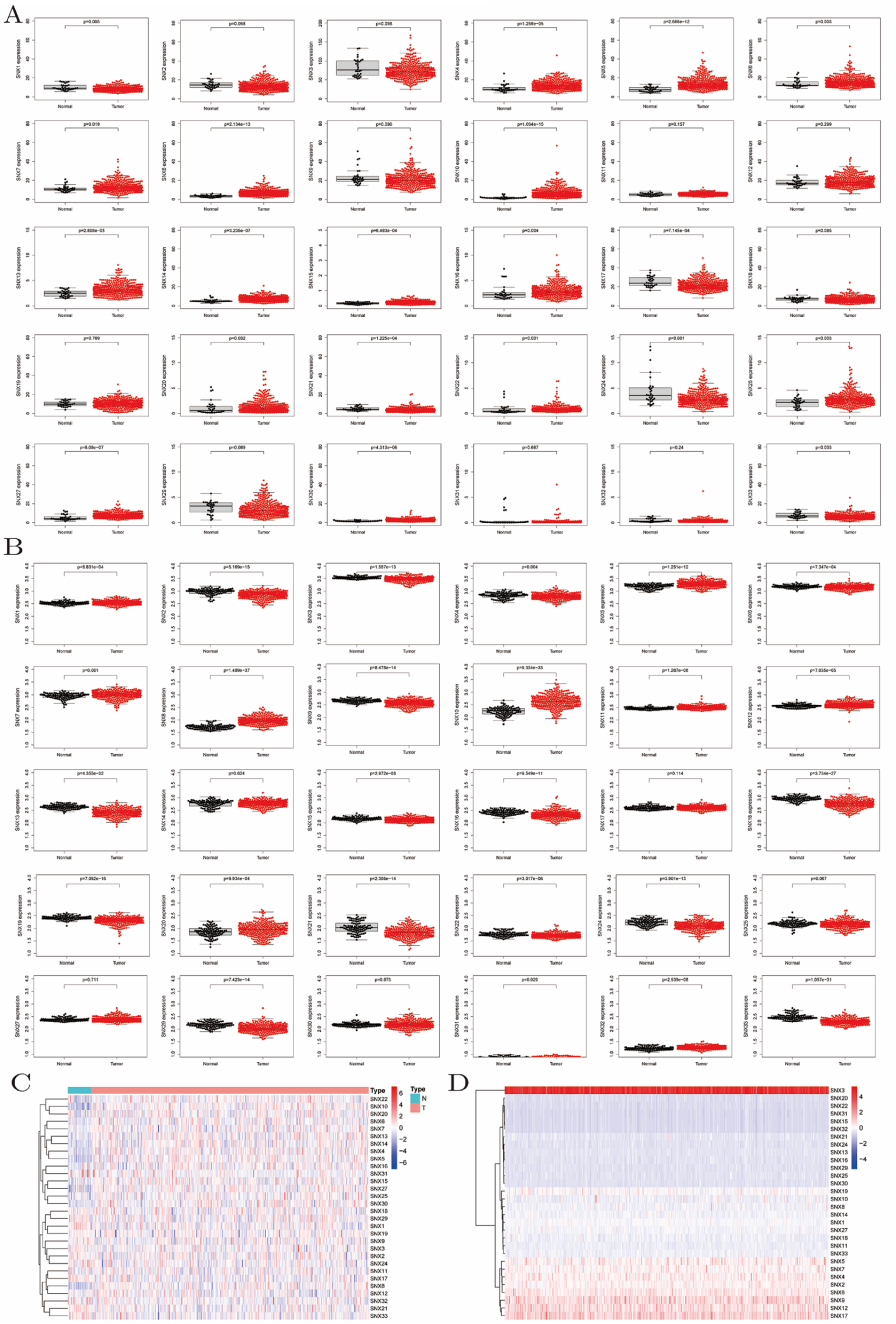
Transcriptional level of SNX family in GC. (**A**) SNX4/5/6/7/8/10/13/14/15/16/17/20/21/22/25/27/30 were higher expressed in tumor, while SNX1/24/33 did the opposite, with no record of SNX23/26/28 in TCGA transcriptome. SNX2/3/9/11/12/18/19/29/31/32 showed no statistic significant in analysis of expression profile. (**B**) Differential expression analysis of 30 SNXs based on GSE66229 as verification, with data of expression profiling by array normalized with log function and thus difference seemed not such apparent. However, statistical significance was still demonstrated in 25 SNXs, SNX23/26/28 not recorded either. (**C**) Clustering analysis of SNXs mRNA between tumor and paired normal cancer manifested the expression profile intuitively. (**D**) Clustering analysis among tumor samples classified SNXs into 6 expression modules.

The result revealed that 15 out of 30 SNXs were significantly higher expressed in GC, including SNX4/5/6/7/8/10/13/14/15/16/1720/21/22/25/27/30, but SNX1/17/21/24/33 were in the opposite expression profiles, other SNXs shown no statistic significant (Fig. 2A). Sequencing data in GSE66229 was normalized with log function by the data provider, and thus transcriptional levels of SNXs were all limited within 1–4, as shown in Fig. 2B. Clustering analysis between cancer and normal tissue showed differentially expression of SNX members intuitively, as shown in Fig. 2C. Corresponding to beeswarms, clustering analysis in Fig. 2D dictates a highest transcriptional level of SNX3, and then SNX2/4/5/6/7/9/12/17, SNX15/20/22/31/32 extremely low expressed and then SNX13/16/21/24/25/29/30, SNX1/8/10/11/14/18/27/33 transcribed at a middle level.

### Internal correlation of the SNX family at transcriptional and translational level

**Figure 3:**
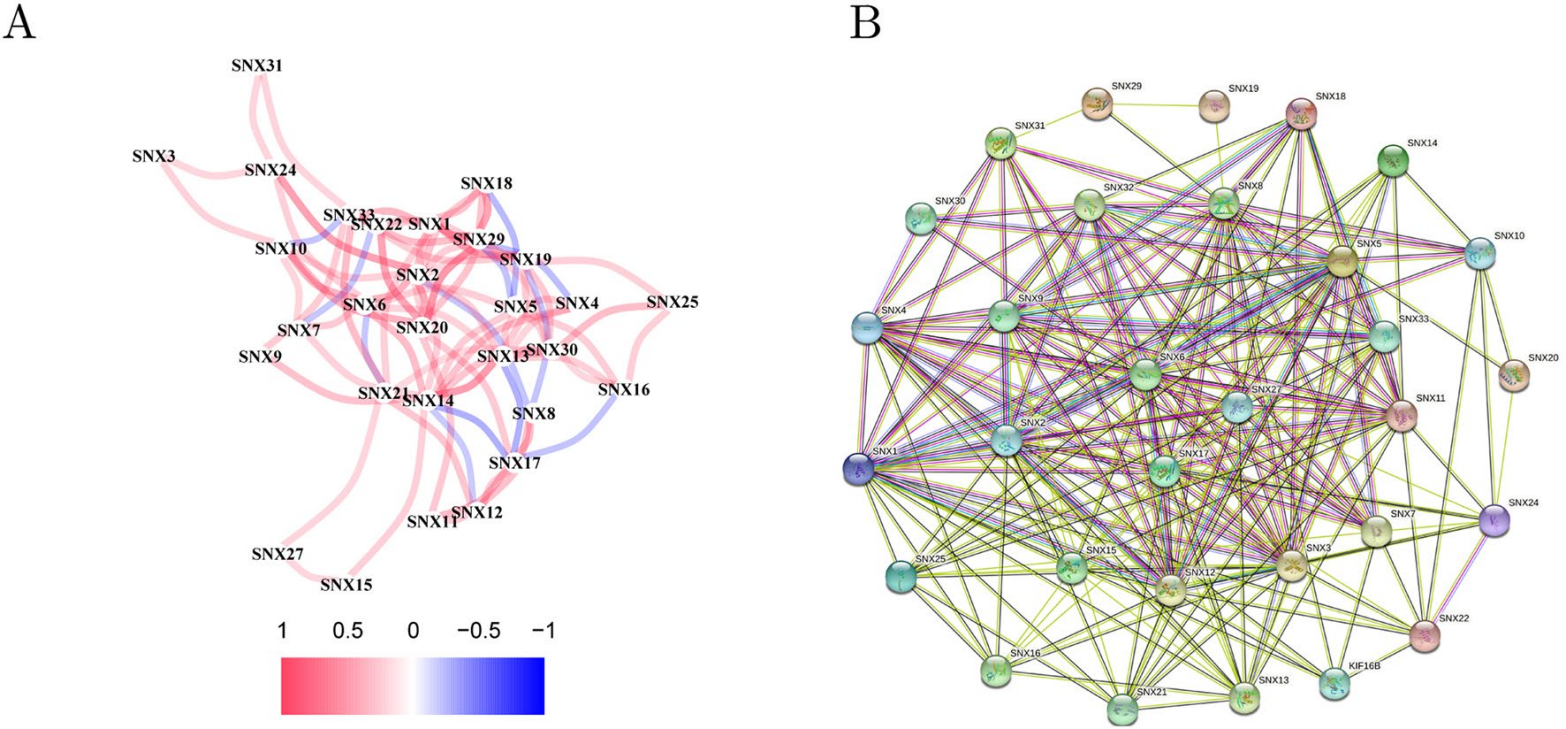
Inner correlation among SNX family at mRNA and protein level. (**A**) Spearman relevance analysis between each SNX member using R language, color red indicating positive correlation while blue did the opposite. (**B**) Protein–Protein Interaction map downloaded from String database, showing that SNXs proteins were all in a well-connected network, SNX23 also called KIF16B, and SNX26/28 were removed from the map for its dissociative connection.

Most SNXs were positively correlated at transcriptional level, with small part of them negatively associated. Corresponding to clustering analysis above, SNXs in negative correlation were in opposite expression profiles, that is to say SNX8/13/14/16/18/21/22/29 were less transcribed than SNX2/4/5/6/7/12/17/19 did in GC (Fig. 3A). PPI declared a close and complicate correlation among SNX proteins. As shown in Fig. 3B, denser connecting lines in purple, black, fluorescent green supposed that most SNX proteins were correlated with each other, evidenced by experiments, co-expression and text-mining, respectively. Except for SNX26 (ARHGAP33) and SNX28 (NOXO1), other 31 SNXs all functioned as connecting nodes in the PPI, even SNX23(KIF16B) involved despite of absence in TCGA database.

### Tumor-infiltrating immune cells and associated SNXs in GC

**Figure 4:**
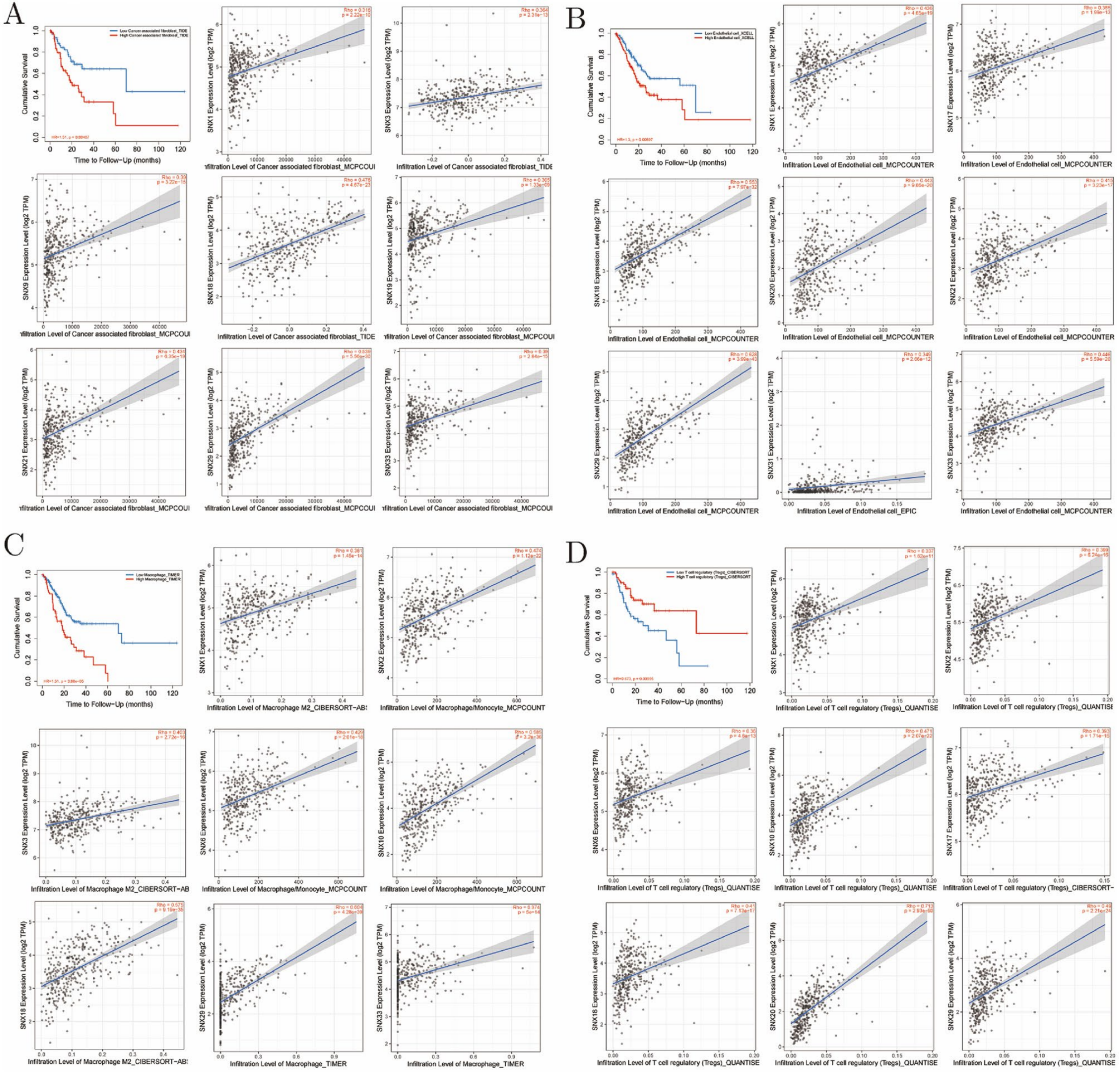
Tumor immune infiltration analysis using cBioportal. Cumulative survival associated tumor infiltrating immune cells (TIICs), cancer associated fibroblast (**A**), endothelial cells (**B**), macrophages (**C**), Tregs (**D**), and top 8 SNXs related to the four TIICs with Spearman coefficients over 0.3.

First, Kaplan–Meier survival plot was portraited to screen prognosis associated TIICs, and patients with higher level of cancer associated fibroblast, endothelial cells, and macrophage infiltration turned to meet a shorter overall survival, while Tregs was on the contrary (Fig. 4). Then, we listed the top 8 SNXs demonstrated to be associated with infiltration level of prognostic TIICs in GC during the Spearmen relevance analysis, other SNXs with Spearman coefficient less than 0.3 presented in supplementary material (Fig. S1). These findings strongly suggested that SNXs play a specific role in immune infiltration in gastric cancer, especially those of macrophages and epithelial cells.

### Role of SNX family in OS, PFS and PPS of GC

**Figure 5:**
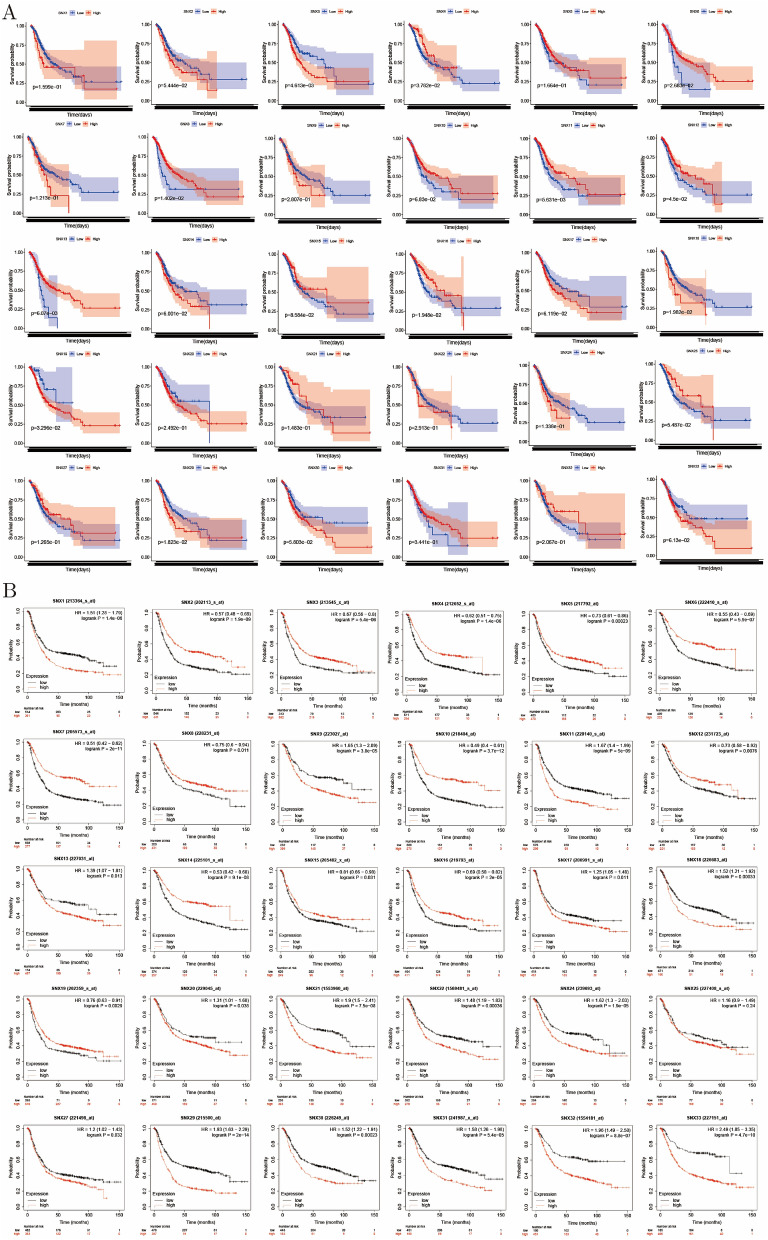
Kaplan–Meier survival analysis of SNX family. (**A**) SNX3/6/8/11/12/13/16/18/19/29/ showed associated with OS of GC based on TCGA clinical information, SNX23/26/28 not recorded. (**B**) GEO online analyzing tool, Kaplan-MeierPlotter, manifesting that 30 SNXs were correlated with OS of GC, with no information of SNX23/26/28 either.

Using clinical data from TCGA and survival R package, correlation between SNXs mRN abundance and OS was elucidated, as shown in Fig. 5A. Surprisingly, 11 out of 30 SNXs were demonstrated to be related to OS in GC, and higher transcriptional level of SNX3/18/19/29 suggested shorter survival time while SNX4/6/8/11/12/13/16 were just in the contrary. Whereas, results from Kaplan–Meier Plotter demonstrated that nearly all the SNXs were of great value in OS except for SNX25(Fig. 5B), in PFS except for SNX12/25, and in PPS except for SNX12/25, SNX23/26/28 excluded either (Fig. S2). No need to emphasize that the lager the sample size is, the more convincing the result is when performing statistical analysis, so we considered the latter result more supportive and the SNX family a crucial role in GC prognosis.

### Mutation of SNX family contributing to malignancy in GC

**Figure 6:**
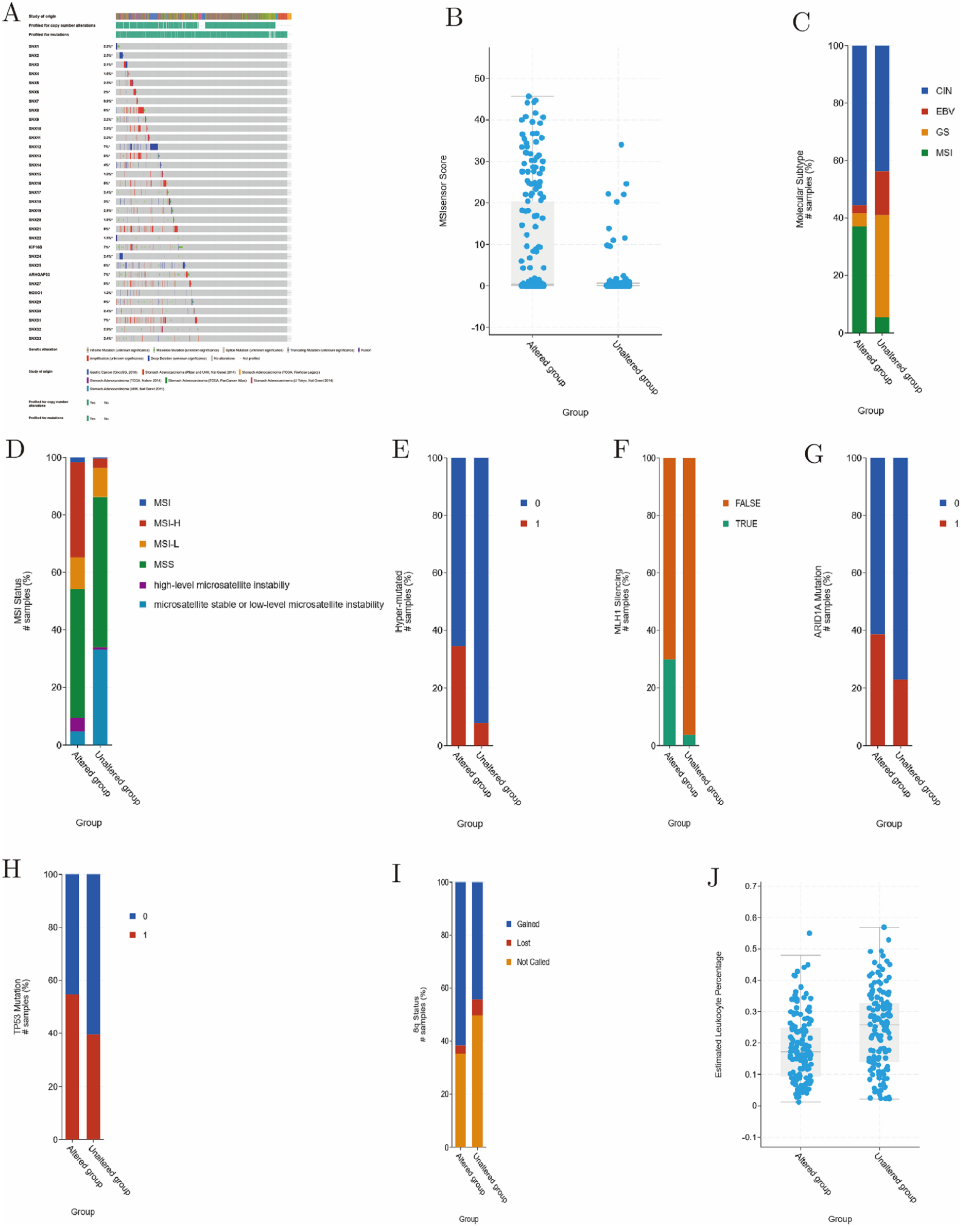
Effects of alteration of SNX family determined with cBioportal. (**A**) Alteration frequency of SNX family in GC, with 700 out of 1512 mutated. SNX family alteration contributing to MSI (**B–C**), high level of MSI (**D–E**), methylation of MLH1(**F**), mutation of tumor suppressing genes (**G–H**), gain of 8q (**I)**, and alleviation of tumor immune infiltration (**J**).

A total of 712 samples out of 1512 (47%) with gastric cancer, most of which were adenocarcinoma, had altered expression levels of at least one of the SNXs (8% of samples with altered expression of SNX21, 7% of samples with altered expression of SNX12/23(KIF16B)/26, 6% of samples with altered expression of SNX16/25, 5% of samples with altered expression of SNX13/27/29, and other SNXs left with alteration frequency less than 5%; Fig. 6A). Additionally, the altered group manifested a higher MSI Sensor score, evidencing the probability of MSI (Fig. 6B; median of 0.33 vs. 0.04; *P* < 10–10). Secondly, the altered group exhibited a higher proportion of MSI (37.09%) than unaltered group (5.56%) did (Fig. 6C; *P* < 10–10). Then, we further investigated detailed status of MSI including three levels of microsatellite stable (MSS), high (MSI-H or hyper-mutated MSI) and low (MSI-L), and patients with SNXs altered held significant higher level of MSI-H than unaltered group did (Fig. 6D); 32.94% vs. 3.24%; *P* < 10–10; Fig. 6E; 34.67% vs. 7.91%; *P* = 8.27–8). All the finding suggested that patients with GC would be more likely to suffer from MSI and MSI-H, given that with SNXs mutating. Intriguingly, methylation silencing of MLH1, evidenced to be mismatch repair gene and stimulus to MSI, was also more commonly seen in the SNXs altered group (Fig. 6F; 30% vs. 3.64%; *P* = 4.49–7). Besides, we found that the altered group exhibited higher alteration frequency of either ARID1A or TP53 than the unaltered group did (Fig. 6G–H; 38.67% vs. 23.02%; *P* = 6.096–3; 54.67% vs. 39.57%; *P* = 0.0143). Additionally, the altered group was more likely to suffer 8q gain mutation (Fig. 6I; 61.54% vs. 44.32%; *P* = 5.881–3). Notably, SNXs mutation exerted negative effect on leukocytes infiltration and might alleviated tumor immunity in GC (Fig. 6J; 0.172 vs. 0.258; *P* = 2.422–5). According to these data, it’s reasonable to draw a conclusion that SNXs alteration might contribute to MSI and many other malignant mutational events in GC.

### A prognostic nomogram based on multivariate analysis

**Figure 7:**
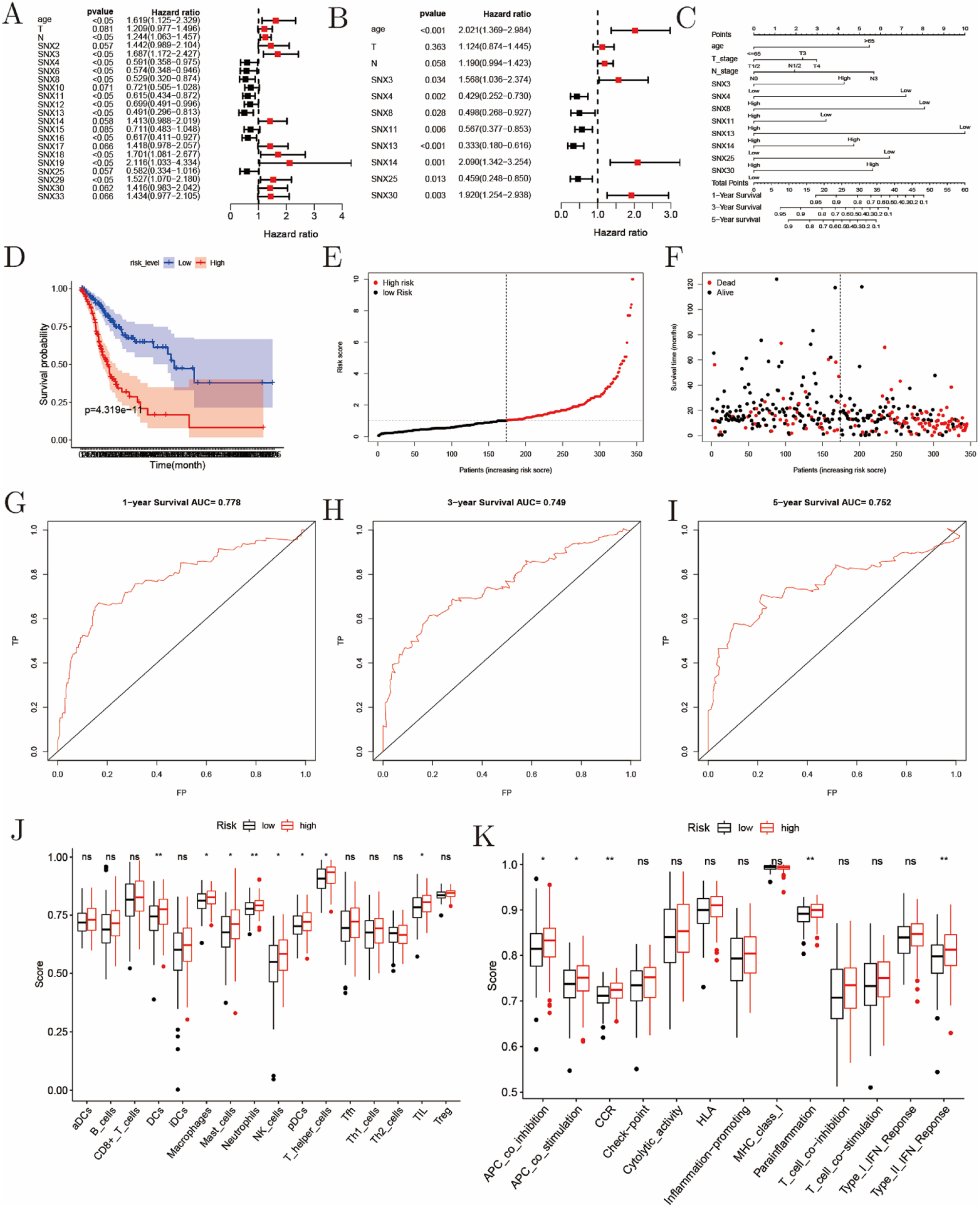
Risk classification model constructed based on multivariate analysis using data in TCGA. (**A–B**) Univariate and multivariate analysis of role of SNXs transcriptional abundance in OS, visualized with forest plots. (**C**) A nomogram to estimate one-, three- and five-year OS. (**D**) KM curve of those with high or low risk judged by the model. (**E**–**F**) Risk distribution plots of all the 345 patients with GC. (**G–I**) Receiver operating curves aiming at one-, three- and five-year OS prediction. (**J–K**) Evidence of co-existence of higher risk and deeper tumor immune infiltration.

As shown in Fig. 7A, SNXs with *P* < 0.1 during univariate analysis were listed in the forest plot, and that SNX4/6/8/10/11/12/13/15/16/25 functioned as beneficial elements for OS while SNX2/3/14/17/18/19/29/30/33 were just on the contrary. Multivariate analysis showed that SNX3/4/8/11/13/14/25/30 were independent risk factors for OS in GC (Fig. 7B). Then, survival-associated variable including age, T-stage, N-stage and SNX3/4/8/11/13/14/25/30 were involved in the nomogram plotting for one-, three- and five-year OS evaluation (Fig. 7C). The accuracy of training set and validation set was 0.75 and 0.72, with proportion set as 6:4. Then the patients were split into two groups by the median of risk-score calculated with survival package based on the model, and those with high risk were more likely to live a shorter OS time than those with low risk did (Fig. 7D). Risk contribution plots showed risk-score of each patient, and those with higher risk-score would encounter shorter survival time and higher mortality (Fig. 7E–F). In addition, using risk-score, we employed ROC to assess value of the model in prognostic prediction, and AUC was 0.778, 0.749 and 0.752, corresponding to one-/three-/five-year OS estimation, respectively (Fig. 7G–I). Finally, patients were averagely split into three groups according to the risk-score rank. As displayed in Fig. 7J–K, those in the high risk-score group hold deeper infiltration of DCs, macrophages, mast cells, neutrophils, NK cells, pDCs, T helper cells, and TIL, and stronger immune cell function of APC co-inhibition, APC co-stimulation, CCR, Para-inflammation, and typeIITFN response. All the findings revealed that SNX3/4/8/11/13/14/25/30 helped make a good model for risk classification of patients with GC.

### ANN models performing excellently for OS and RFS prediction

**Figure 8:**
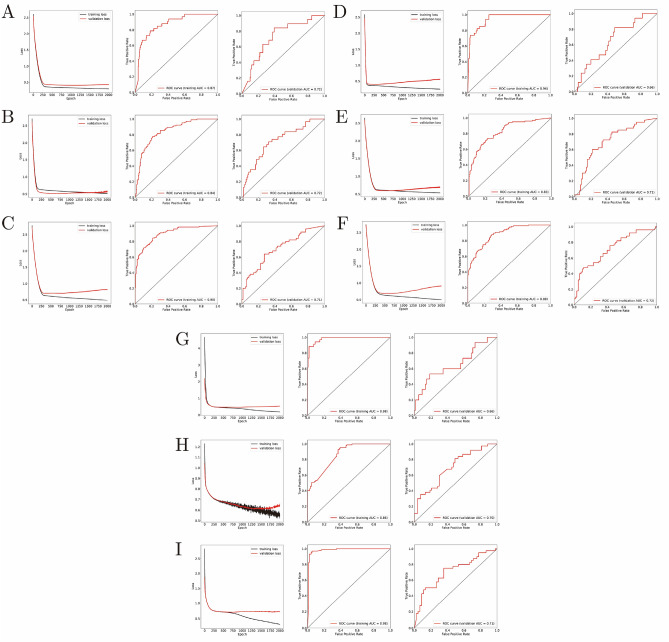
Efficiency estimation of six ANN predictors. (**A–C**) Loss curve, ROC of training set and validation set of models aiming at one-, three- and five-year OS prediction based on GSE84437. (**D–F**) Loss curve, ROC of training set and validation set models aiming at one-, three- and five-year RFS assessment based on GSE26253. (**G–I**) Loss curve, ROC of training set and validation set models aiming at one-, three- and five-year RFS assessment based on GSE66229, with only transcriptional data of SNX family members.

As shown in Fig. 8A, loss curve of both training set and validation set came down to a smooth level, indicating that the model had already performed its predicting capability at its peak after 2000 times iteration. AUC of training set and validation set in the first model was 0.87 and 0.72, respectively. Except for the first model, loss curves of the left five model were not smooth enough, but they still reflected satisfactory prognostic ability. As shown in Fig. 8B–I, AUC of training set and validation set were 0.84 and 0.72, 0.90 and 0.71, 0.94 and 0.66, 0.83 and 0.71, 0.88 and 0.72, 0.98 and 0.66, 0.86 and 0.70, 0.98 and 0.71 respectively.

## Discussion

SNX protein family is broadly distributed in cytoplasm especially the endosomal network, and plays a great part in sorting and transportation of cargo protein in the endomembrane system of eukaryotes, keeping going cell biosynthesis and material secretion. To our knowledge, the study was the first to discuss the correlation between SNX family and gastric cancer prognosis using bioinformatics technology comprehensively, taking SNX family as a whole. Expression profiles of SNX family and relationship between transcriptional abundance and survival rates, association between SNX mRNA expression and tumor immunity, relevance between SNXs alteration and MSI status as well as tumor suppressing genes mutation were all included in this study, and finally one multivariable cox regression model for risk classification and six prognostic models based on ANN were employed to further disclose the prognostic role of SNX family in GC.

Based on TCGA gastric cancer transcriptome data STAD, we identified differentially expressed genes in gastric cancer comparing to adjacent normal tissue. SNX4/5/6/7/8/10/13/14/15/16/20/22/25/27/30 were overexpressed in gastric cancer, while SNX1/17/21/24/33 were higher expressed in normal tissue. Although results from GSE66229 was a bit different from the TCGA cohort, there were still 25 SNX members evidenced to be differentially expressed, and we guessed that the discrepancy might come from the normalization with log function or another USA cohort. In addition, Kaplan–Meier survival analysis based on TCGA showed that high expression of SNX3/18/19/29 indicated a shorter OS period, while those with SNX4/6/8/11/12/13/16 low expressed lived longer. KM curves presented with Kaplan–Meier Plotter revealed that much more SNX family members were closely related to OS, PFS, and PPS. It was displayed that higher expression of SNX1/9/11/13/17/18/20/21/22/24/27/29/30/31/32/33 predicted a shorter OS, SNX2/3/4/5/6/7/8/10/12/14/15/16/19 just did the opposite. High translational level of SNX1/9/11/13/17/18/20/21/22/24/27/29/30/31/32/33 implied worse PFS, while SNX2/3/4 /5/6/7/8/10/14/15/16/19 did the opposite. PPS analysis gave the same result as PFS analysis did. Generally, analysis based on KM-Plotter offered a more satisfactory result than that based on TCGA did, because of a larger sample size, ranging from 631 to 875. Secondly, SNX25 was proved to be no statistically significant in the survival analysis based on neither TCGA and GEO database, and SNX12 did the same in FP or RFS analysis. What's more, we found an intriguingly phenomenon that SNX/4/5/6/7/8/10/13/14/15/16 is highly expressed in GC, but high expression suggested a better prognosis. SNX1 was lower expressed in gastric cancer, but its lower expression associated with longer survival time. Such results challenged we researchers’ conventional cognition, but it might also reflect the complexity of tumor prognostic study on the other hand. Actually, there have already been some, but not many members of the SNX family documented to be related to initiation, progression and prognosis of several cancer types. SNX1 has been deeply studied in gastrointestinal carcinoma. SNX1 mRNA and protein were first demonstrated to be low expressed in colon cancer, and gastric cancer cells with SNX1 deletion showed stronger proliferation ability and were more likely to activate the signal transduction of EGFR-ERK1/2 pathway induced by EGF, along with the sensitivity to anoikis decreased^17^. Then, miR-95 was found to bind to the 3'untranslated region of SNX1, and promoted proliferation of colon cancer cells caused by miR-95 overexpression could be reversed by SNX1 overexpression, suggesting that miR-95 alleviated anticancer effect of SNX1 in colon cancer^18^. Consistent with our study, SNX1 has also been shown to be lower expressed in gastric cancer by Xiao-yong Zhan et al. but patients with SNX1 high expression harbored longer OS, which is inconsistent with this study^10^. In gefitinib-resistant non-small cell lung cancer cells, SNX1 was found to inhibit the endocytosis and degradation of MET whose overexpression was believed to be responsible for gefitinib resistance in non-small cell lung cancer^19^. Similarly, regulating the degradation of c-MET in lysosomes, SNX2 was expected to be a novel drug target to elevate the sensitivity to EGFR-targeted drugs in non-small cell lung cancer^20,21^. SNX2 might play a tumor suppressing role in liver cancer and colon cancer. SNX2 deletion has been found to promote hepatocyte growth factor receptor tyrosine phosphorylation and activation of ERK1/2 pathway. At the same time, SNX2 was lower expressed in colon cancer, and it suggested smaller cumulative survival rate^22^. Again, in high-grade gliomas, the overexpression of SNX3 disrupted EGFR and MET endosomes, inhibited the degradation of both through lysosome lysis, and thus promoted the proliferation of gliomas^23^. SNX5 is one of the components of the mammalian cargo-selective complex of retromer, so SNX5 exerted impact on tumor progression by directly affecting the transportation of diverse cell surface receptors or others. High expression of SNX5 was demonstrated in well-differentiated papillary thyroid carcinoma, and co-expression of SNX5 and caspase-2 was also found in thyroid epithelial cells^24^. Meanwhile, high level of TSH was commonly considered to be a risk factor for recurrence of thyroid cancer after surgery, and SNX has been shown to suppress TSH expression^25^. However, there were also reports asserting that SNX5 was accused of inhibiting the degradation of EGFR, and this mechanism was later confirmed in hepatocellular carcinoma^8,9^. Similarly, SNX5 bound to FBW7, thereby indirectly distract FBW7 from interacting with oncoproteins such as MYC, NOTCH and Cyclin E1 to mediate their degradation by ubiquitination, leading to an increase of oncoproteins and promoting the progression of head and neck squamous cell carcinoma^26^. As also institutional structure of retromer, SNX6 has been reported to enhance the core effect of breast cancer transcription in a dose-dependent manner, that is suppressing transcription in breast cancer^27^. In pancreatic cancer cells, SNX6 overexpression has been witnessed to maintain mesenchymal properties of tumor cells, contributing to metastasis, while SNX6 silencing inhibited the EMT process induced by TGF-β, suggesting engagement of SNX6 with metastasis of pancreatic cancer^28^. SNX9 has been shown to lower expressed in breast cancer and non-small cell lung cancer in highly advanced stage. Besides, SNX9 was co-localized with TKS5, a marker of invasive pseudopodia, and overexpression of SNX9 negatively regulated the number and function of invasive pseudopodia, thereby reducing its extracellular degradation^29^. In addition, overexpression of SNX9 has been found in vascular endothelial cells in colon cancer, which was proved to be associated with poor prognosis of colon cancer. At the same time, SNX9 was regarded as a new vascular regulator, because SNX9 knockout would decrease the recycling rate of β-integrin, resulting in a smaller amount of β-integrin on cell surface^30^. SNX10 manifested a tumor suppressing influence by regulating autophagy behavior of tumor cells to inhibit the progress of colorectal cancer^31–33^. SNX27 is a special member of the SNX family. In addition to the PX domain, it also contains a PDZ domain. G protein-coupled receptors are the largest membrane protein family and are broadly engaged with transduction of multiple intracellular downstream signaling pathways. Binding to the PDZ binding motif of G protein-coupled receptors through PDZ domain, SNX27 interferes with its recycling from the endosome to cell membrane, and thus SNX27 is expected to be the next promising tumor therapeutic target^34^. In general, SNX family members participating in construction of the retromer complex were directly involved in the degradation or cycling of numerous receptors, but other SNXs were also illustrated to regulate intracellular transportation through their distinct domains such as PDZ domain, giving an insight into the reason why aberrant expression of SNX family members affects tumor prognosis. Given that multitudes of bioinformatical analysis have been conducted, we think there is still some can be done during this part. Differential expression is limited at mRNA level, and thus Western Blot for SNXs protein detection should be performed in GC cells and normal gastric mucosal cells, GC specimens and para-tumorous tissue in future.

Analysis using Timer showed that cancer associated fibroblasts, endothelial cells, macrophages and Tregs were statistically significant in the survival analysis.SNX1/3/9/18/19/21/29/33,SNX1/17/18/20/21/29/31/33,SNX1/2/3/6/10/18/29/33, and SNX1/2/6/10/17/18/20/29 were strongly correlated with TIICs mentioned above, with Spearman coefficients all over 0.3. SNX29 seemed to be the next research hotspot of immune regulation in gastric cancer, for its positive correlation with all the four TIICs types. Although the latest study showed that SNX5 mediated autophagy and immunity induced by virus infection, there has been few reports of immune-related research on SNX family^35^. SNX4-SNX7 heterodimers has been verified to recruit autophagy regulators in the early stage of autophagosome assembly, and that SNX4 knockout will cause failure of rapidly ATG9A transportation from the perinuclear to the autophagosome-assembling site upon stimulation of autophagy to form the peripheral membrane pools necessary for autophagy assembly^36^. In addition, SNX18 has also been documented to interact with Dynamin-2 to induce membrane budding of recycling endosomes containing ATG9A and ATG16L1, which were then transported to the place where autophagosomes would be formed to participate in autophagosome assembly^37^. As mentioned above, in colon cancer, SNX10 has been proved to regulate expression of a core effector, P21, in tumor suppressing pathways and to affect metabolism of amino acids mediating activation of mTOR by regulating chaperone-mediated autophagy. Lacking for SNX10 would lead to reduced SRC endosomal lysosomal degradation, thereby activating SRC-mediated STAT3 and CTNNB1 signaling pathways^31–33^. These data indicated that the SNX protein family might be more likely to participate in tumor immunity in an indirect way through regulation of autophagy.

The concept of MSI was put forward by Z LODIN in the central nervous system in 1958, but it was since 1991 that people started disclosing its specific role in tumor initiation, progression and even prognosis^38^. The mismatch repair mechanism, once impaired leading to MSI occurrence, is mediated by various mismatch repair enzymes, including MLH1 whose promoter methylation directly leads to MSI occurrence^39^. The first type of MSI encompasses MSS (microsatellite stable), MSI-H (microsatellite instability-high), and MSI-L (microsatellite instability-low); the second type refers to MSI-H, MSS/MSI-L, and data of MSI status in this study from cBioportal database applied both of them^40, 41^. The mutation frequency of the SNX family reached 47%, and the altered population was more likely to suffer from MSI and MSI-H or hyper-mutated MSI. Consistent with the result mentioned above, the altered samples were detected with higher frequency of MLH1 silencing, or MLH1 promoter methylation in other words. AT-Rich Interaction Domain 1A (ARID1A), characterized as chromatin remodeling gene, and TP53, are broadly considered as tumor suppressors, both more commonly seen mutated in SNXs altered group. Corresponding to prior reports, deficiency of mismatch repair mechanism arising from MLH1 promoter methylation has been proved a stimulus to MSI, and ARID1A mutation were more commonly seen in those with MSI, suggesting ARID1A Mutation might also be a contributor to MSI^42^. In addition, the altered group had a higher probability of 8q gain, and studies have confirmed that SNX8q gain functioned as a negative predictor of prognosis in prostate cancer, renal clear cell carcinoma, resectable pancreatic adenocarcinoma, and hematological malignant tumors^43–45^. It has been documented that C-MYC was located at 8q and 8q gain might up-regulate the expression of C-MYC, resulting in activation of downstream MAPK/ERK pathway^46^. Therefore, we here conclude that SNX family alteration may contribute to various malignant mutational events such as that of ARID1A, TP53 and MLH1, thus leading to MSI in GC, but there needs further research for winnowing out SNXs that playing the biggest role.

Through univariate and multivariate analysis, we found that characteristics of age, SNX3/4/8/11/13/14/25/30 were independent risk factors for OS in GC. From the nomogram, we found that SNX4/8/13 had greater impact on risk classification for patients even than T/N-stage did. The C index of the training set and the validation set divided by a ratio of 6:4 was 0.75 and 0.72, respectively, and AUC were 0.778, 0.749, and 0.752. The model manifested promising potential for risk classification, for those defined as with high risk underwent apparent shorter survival period and higher mortality. Finally, high risk acted as herald of higher immune cells score and higher immune cells function score, which again proved the positive relevance between SNX family expression and tumor immune infiltration. These findings suggested that the nomogram established with SNXs had nom-negligible potential of risk stratifying and OS predicting for patients with GC. Here value of SNXs in OS evaluation was dissected with multivariate cox regression, and relation between SNX proteins and other clinicopathological parameters remained unclear. We will first collect 200–300 GC specimens from Northeastern of China under patients’ permission, and then protein level of SNXS will be detected with semi-quantitative analysis, immunohistochemistry, following investigation of effects of diverse protein level of SNX members on clinicopathological characteristics encompassing age, c-TNM stage, p-TNM stage, vascular invasion, lymphatic vessel invasion, tumor counts, tumor size, grade and differentiation.

With SNX3/4/6/8/11/12/13/16/19/29, age, T-stage, and sex as feature values for OS estimation, SNX3/6/8/11/12/13/19 and pathological stage inputted as feature values for RFS evaluation, six ANN models were established as verification based on another two Korean cohorts. AUC of training sets and validation sets in one-/three-/five-year OS/RFS prediction models was 0.87/0.72, 0.84/0.72, 0.90/0.71, 0.94/0.66, 0.83/0.71, 0.88/0.72. The one-year RFS prediction model seemed not valuable enough, and it might be caused by the unbalance of 428 samples within which recurrence accounted for only 11.7%, leading to less outstanding generalization ability of the model. Besides, GSE66229 with 300 GC patients’ information was also included for another three ANN model construction for OS prediction, with AUC corresponding to one-/three-/five-year of 0.98/0.66, 0.86/0.70, 0.98/0.71. The one-year ANN model seemed not satisfactory enough, for there were only 50 patients died within one year and thus the artificial neural network lacked enough information during training progress. Actually, the ANN models built with GSE66229 are supposed to be the most convincing verification to the nomogram model, because the input of them encompassed only SNX members, without other clinicopathological characteristics in other words. Concerning to the approaches of building models, TCGA cohort was employed for a multivariate cox regression model visualized as the nomogram, because there was quite a big part of censored data in the survival following-up information, which makes it hard to establish ANN models after patients with censored data were removed because of lacking for adequate samples. Whereas, GSE84437 and GSE26253 cohorts from Korea were adopted as input of ANN models, because there were still more than 400 samples respectively after censored data was removed before model building. Taking all the findings into accountant, we here demonstrated the eminent role of SNX family in prognosis estimation of GC, but the detailed molecular mechanism underlying that needs further research. In future, we will first perform SNXs knockdown/overexpression experiments and observe its following effects on proliferation, migration and invasion, apoptosis, and colony formation. According to documented reports, SNXs have been proved participating in tumor progression through protein transporting, and thus SNX-BAR sub-family members involved in retromer constitution will be the next focus in the following experimental research in order to uncover the potential molecular mechanism in GC.

## Conclusion

We here asserted that at least 20 SNX family members were differentially expressed in GC, and 29 SNXs were demonstrated associated with OS, PFS and PPS. Additionally, SNX family alteration contributed to MSI and mutation of tumor-suppressing genes encompassing MLH1, ARID1A and TP53. Risk classification model constructed using clinical characteristics like age, T-stage and N-stage, as well as SNX family members based on TCGA cohort distinguished patients with high or low risk effectively. A nomogram based on the risk classification model displayed high accuracy during post-operation OS evaluation. ANN models based on another three cohorts from GEO aiming at short-term and long-term OS and RFS prediction performed excellently as convincing verifications of the TCGA American cohort, uncovering the prognostic role of the SNX family in GC.

## Supplementary Information

Supplementary Information.

## Data Availability

All data and code included are available upon request by contact with the corresponding author. Supplementary materials can be found online.
