# Supplementary material for: Identification of specific role of SNX family in gastric cancer prognosis evaluation
Source: Sci Rep. 2022 Jun 17;12:10231. doi: 10.1038/s41598-022-14266-y (PMC9205943; doi:10.1038/s41598-022-14266-y)
Supplement: Supplementary file 1 — Supplementary Information. [file 41598_2022_14266_MOESM1_ESM.docx]

**Figures and figure legends**


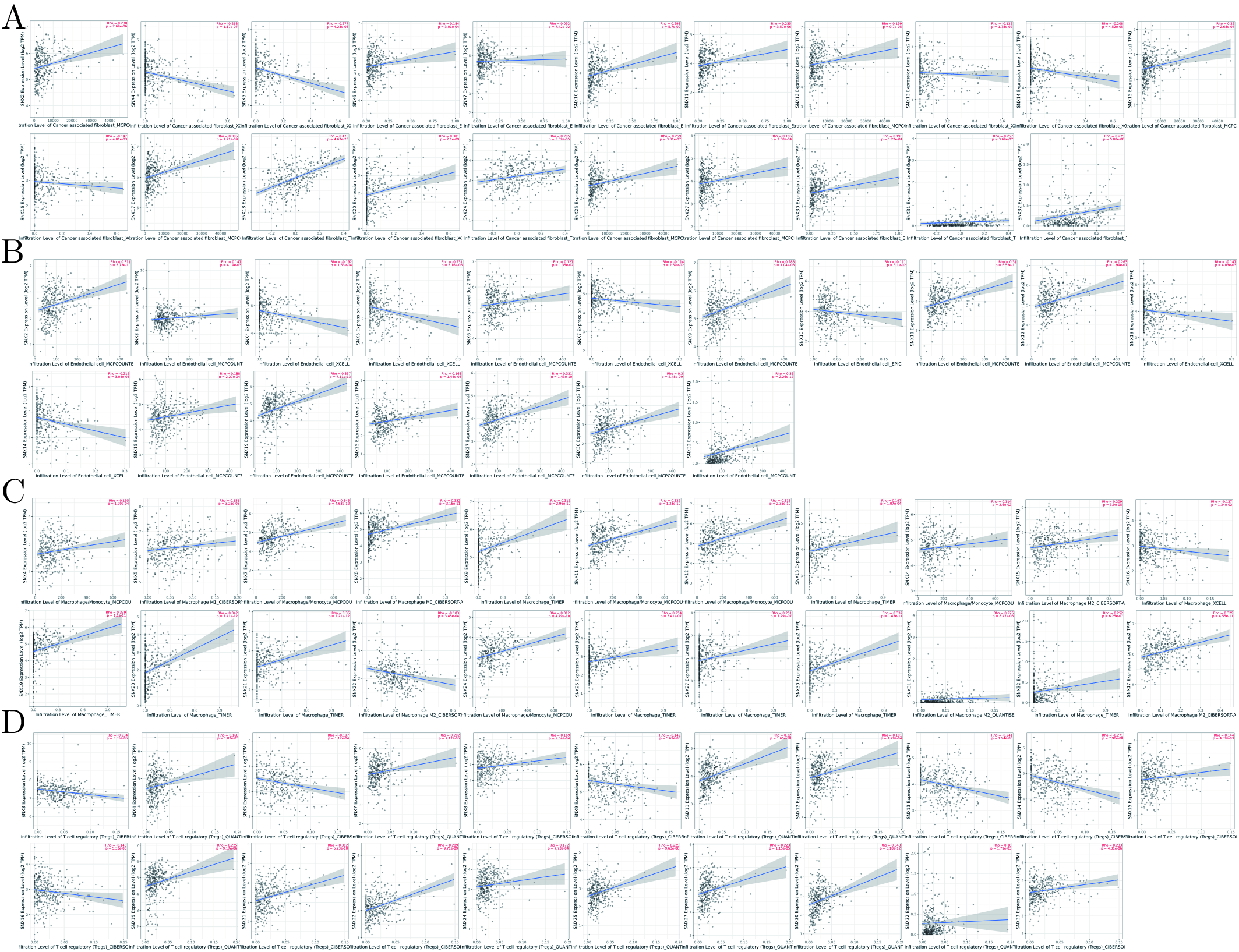


**Figure S1.** Spearman relevance between SNX family members and four types of tumor-infiltrating immune cells including cancer-associated fiber cells(**A**), endothelial cells(**B**), Macrophages(**C**) and regulatory T cells(**D**). Except for SNX 23/26/28 not recorded in Timer database, other SNXs with P<0.05 are all presented.


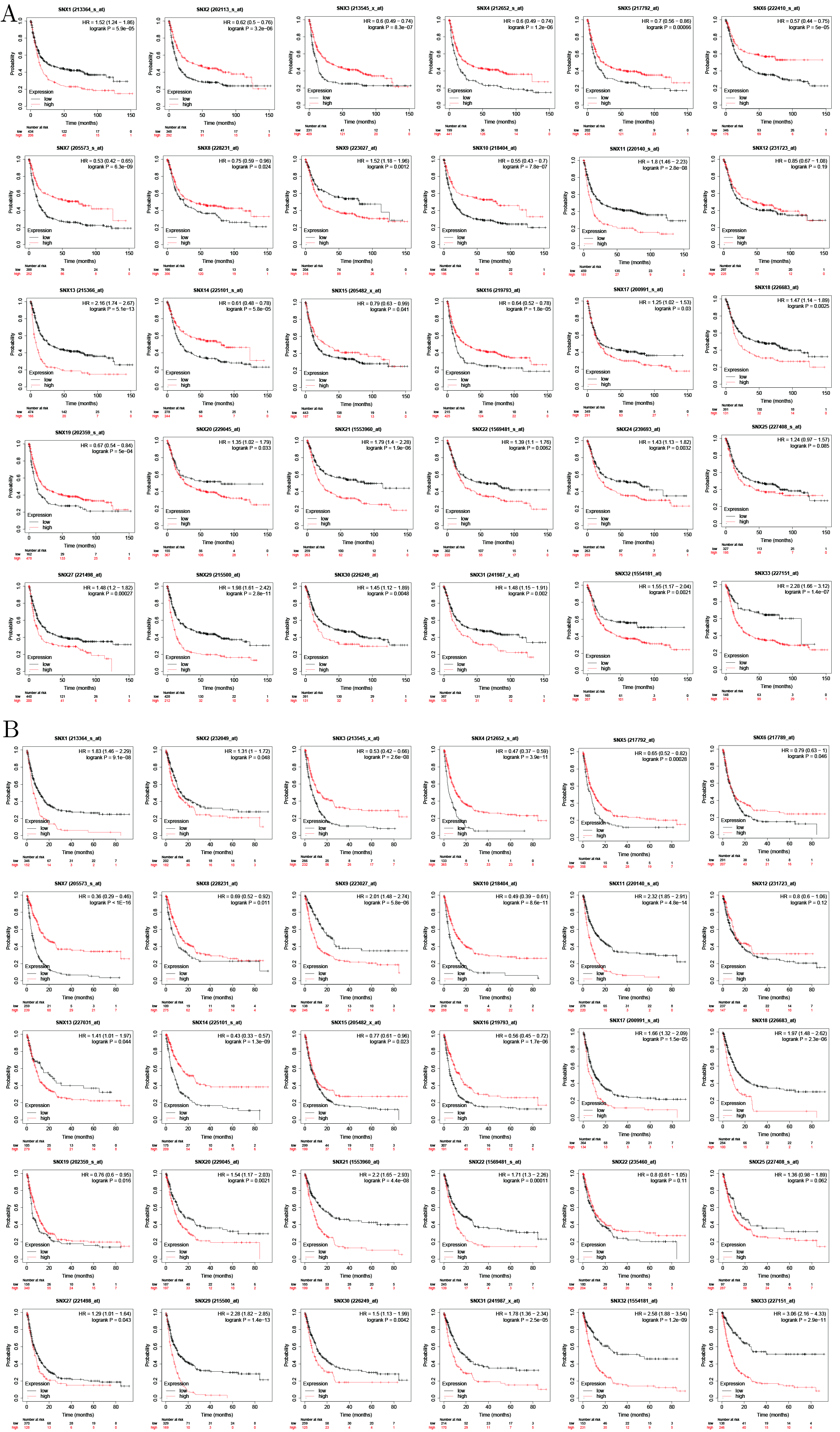


**Figure S2.** Kaplan-Meier survival analysis between 30 SNXs and PFS/PPS. Using KM-Plotter , a GEO online analysis tool, 28 SNX family members were all found to be associated with PFS(**A**) and RFS(**B**) of GC. SNX12/25 shown as no statistic significant, and SNX23/26/28 not recorded either.
